# Photo-Responsivity Improvement of Photo-Mobile Polymers Actuators Based on a Novel LCs/Azobenzene Copolymer and ZnO Nanoparticles Network

**DOI:** 10.3390/nano11123320

**Published:** 2021-12-07

**Authors:** Domenico Sagnelli, Marcella Calabrese, Olga Kaczmarczyk, Massimo Rippa, Ambra Vestri, Valentina Marchesano, Kristoffer Kortsen, Valentina Cuzzucoli Crucitti, Fulvia Villani, Fausta Loffredo, Carmela Borriello, Giuseppe Nenna, Mariacristina Cocca, Veronica Ambrogi, Katarzyna Matczyszyn, Francesco Simoni, Lucia Petti

**Affiliations:** 1 Institute of Applied Sciences and Intelligent Systems of CNR, 80072 Pozzuoli, Italy; marcellacalabrese.97@libero.it (M.C.); olga.kaczmarczyk@pwr.edu.pl (O.K.); massimo.rippa@isasi.cnr.it (M.R.); ambra.vestri@isasi.cnr.it (A.V.); valentina.marchesano@isasi.cnr.it (V.M.); f.simoni@photomat.it (F.S.); 2 Department of Chemical, Materials and Production Engineering, University of Naples Federico II, 80125 Naples, Italy; veronica.ambrogi@unina.it; 3 Advanced Materials Engineering and Modelling Group, Faculty of Chemistry, Wroclaw University of Science and Technology, Wybrzeze Wyspianskiego 27, 50-370 Wroclaw, Poland; katarzyna.matczyszyn@pwr.edu.pl; 4 School of Chemistry, University of Nottingham, University Park, Nottingham NG7 2RD, UK; Kristoffer.kortsen@nottingham.ac.uk; 5 Department of Chemical and Environmental Engineering, University of Nottingham, University Park, Nottingham NG7 2RD, UK; valentina.cuzzucolicrucitti1@nottingham.ac.uk; 6 ENEA, Italian National Agency for New Technologies, Energy and Sustainable Economic Development, Portici Research Centre, Portici, 80055 Naples, Italy; fulvia.villani@enea.it (F.V.); fausta.loffredo@enea.it (F.L.); carmela.borriello@enea.it (C.B.); 7 Institute for Polymers, Composites and Biomaterials of CNR, 80072 Pozzuoli, Italy; mariacristina.cocca@ipcb.cnr.it

**Keywords:** actuators, photo-mobile materials, ZnO nanoparticles, azobenzene monomers, smart materials, liquid crystals

## Abstract

The efficiency of photomobile polymers (PMP) in the conversion of light into mechanical work plays a fundamental role in achieving cutting-edge innovation in the development of novel applications ranging from energy harvesting to sensor approaches. Because of their photochromic properties, azobenzene monomers have been shown to be an efficient material for the preparation of PMPs with appropriate photoresponsivity. Upon integration of the azobenzene molecules as moieties into a polymer, they act as an engine, allowing fast movements of up to 50 Hz. In this work we show a promising approach for integrating ZnO nanoparticles into a liquid crystalline polymer network. The addition of such nanoparticles allows the trapping of incoming light, which acts as diffusive points in the polymer matrix. We characterized the achieved nanocomposite material in terms of thermomechanical and optical properties and finally demonstrated that the doped PMP was better performing that the undoped PMP film.

## 1. Introduction

Photomobile polymers (PMPs) are elastomer films that deform while irradiated with light of the proper wavelength and are controlled by modulating its intensity, polarization state and wavelength.

PMP actuators change their shape or volume when exposed to light [1] because of photo-responsive moieties [2]. Some of the most often investigated photoresponsive liquid crystalline elastomers and thermoset polymers are based on photochromic azobenzene molecules [3,4,5]. The working principle of this molecule is based on photo-induced isomerization between trans (E) and cis (Z) configurations [6,7]. Such photoisomerization causes an isothermal change from an ordered phase to an isotropic phase in a liquid crystal-based matrix, which results in contraction of the material [8]. 

Because of their unique properties, liquid crystalline-based PMPs [9,10] have been under intensive research as potential materials for actuators [5,11,12] and soft robotics [13,14,15] applications.

In principle, liquid crystal molecules (LCs) can exhibit an orientational order [16,17]. Such an order can be induced mechanically by creating grooves on the substrate where the LCs can anchor [18], or by using magnetic and electric fields. The specific LC’s alignment can be preserved by performing a polymerization at the mesophase temperature, creating an ordered polymer network [5,19,20,21,22]. 

The responses of PMPs depend on various factors such as the LC mixture [23], cross-linker concentrations [24], orientation of the molecules [25] and light polarization [26,27]. Currently, composition improvements are going towards decreasing azobenzene moieties in the polymer because of the high costs of the relative monomers. This can be achieved by combining LCs with the azobenzene derivatives [28,29]. This approach provides stable monomer mesophases in a wide temperature range, better penetration of the light through the material and photoresponsivity [30]. 

The addition of nanoparticles (NPs) is an appealing approach to introduce distinct features to the liquid crystals. The NPs’ integration influences the structure’s stability [31], magnetic [32] and electro-optical [33] properties of the material, and can enhance or change the liquid crystals’ own properties [34]. For example, the nanomaterials can modulate the PMP movements by increasing the temperature induced by light (the photo-thermal effect) [35,36]. The simultaneous use of both photochemical and photothermal effects helps to achieve reconfigurable, complex shape changes and enables the use of longer, less damaging wavelengths [29]. Interesting nanomaterials are ZnO nanoparticles, which are semiconductors characterized by a wide band-gap [37], emission peaks in both UV and VIS regions [38] and good heat conductivity [39]. They found diverse applications such as optoelectronics devices, photocatalysis, cosmetics and biosensing [38,40,41], and have already been used to increase the thermal conductivity [42] of the polymers, maintaining their mechanical properties at the same time. However, the major limitation is the aggregation of nanoparticles in the matrix [43] which results in phase separation [44]. Proper functionalization [45] or the choice of specific shapes and types [46] of nanomaterials seem to be the main approaches to avoid this problem [47]. In literature, it possible to find the first attempts in integrating azobenzene and ZnO. Shah et al. (2012) found that a carboxylated azobenzene compound would self-assemble on ZnO nanocrystals, implementing its photochromic properties [48]. Another example would be the production of ZnO: azobenzene superlattice thin films fabricated from diethylzinc, water, and 4,4′-azobenzene dicarboxylic acid precursors [49]. As far as the authors’ are aware, in the literature, there is not yet any report about the integration of ZnO nanoparticles inside an LC-based thermoset. We present a novel photomobile thermoset prepared by mixing LC-based monomers and ZnO nanoparticles with an average length of 100 nm. The doped and bare PMPs were characterized to find out whether mechanical, speed and bending properties of the PMP would improve. In particular, we explored the possibility of synthesizing more efficient and mechanically-improved PMPs, also with the absence of an orientational organization of liquid crystals due to the nanoparticles. This achievement might result in remarkably efficient PMP realization, while avoiding the alignment steps. 

## 2. Materials and Methods

### 2.1. Materials

LC monomers 4-Methoxybenzoic acid 4-(6-acryloyloxyhexyloxy)phenyl ester (MAPE), 4[4[6-Acryloxyhex-1-yl)oxyphenyl]carboxybenzonitrile (AOCB), 1,4-Bis-[4-(6-acryloyloxyhexyloxy)benzoyloxy]-2-methylbenzene (AOBM), 4,4′-Bis[9 -(acryloyloxy)nonyloxy]azobenzene (A9ZA9) were acquired at Synthon Chemicals (Wolfen,, Germany), and the photoinitiator Bis(2,4,6-trimethylbenzoyl)-phenylphosphineoxide from Sigma Aldrich (St. Louis, MO, USA). Elvamide was provided by Beamco (Supplementary Figure S2). The ZnO nanopowder was bought from Sigma Aldrich (Aldrich Cod 544906—average particle size 71 nm—specific surface area 15 m^2^/g).

### 2.2. Methods

#### 2.2.1. Dynamic Scanning Calorimetry (DSC)

Differential scanning calorimetric analysis was performed using a TA-Q2000 differential scanning calorimeter (DSC, New Castel, DE, USA) equipped with an RCS-90 cooling unit (TA Instruments). The instrument was calibrated in temperature and energy with pure indium. For all measurements, approximately 3 mg of sample in the Tzero aluminium pans was analysed at a constant nitrogen flow rate of 20 mL/min. For the evaluation of the main phase transitions, samples were heated from 0 to 200 °C at a rate of 3 °C/min, melted at 200 °C for 3 min to erase previous thermal history, then cooled to 0 °C at 1 °C/min. Finally, samples were heated again at 3 °C/min up to 200 °C. The enthalpy of transitions was calculated by integrating the transition peaks using the software TA Universal Analysis 2000, Version 4.7A. For glass transition evaluation, all the samples were heated from −50 °C to 150 °C, held at 150 °C for 3 min, cooled to −50 °C, and finally reheated at 150 °C. The heating and cooling rates were 20 °C/min. The glass transitions were calculated using the software TA Universal Analysis 2000, Version 4.7A [50].

#### 2.2.2. Thermal Gravimetric Analysis (TGA)

Thermogravimetric analysis (TGA) was carried out on a Pyris 1 TGA analyzer (PerkinElmer, Waltham, MA, USA) about 5 mg of samples were placed in an open platinum pan and heated from 30 to 800 °C at a rate of 10 °C/min using air as purge gas (flow rate: 40 mL/min).

#### 2.2.3. Order Parameter Calculation

In order to evaluate the orientational order of the mixture of LC monomers, we estimated the order parameter using linear dichroism measurements by transmission of light through the sample with a set-up composed of: a polarized laser (wavelength 633 or 457 nm), a heater chamber with an optical window (Mettler Toledo FP82HT, Columbus, OH, USA), and a power detector (Coherent Model M-2). In order to evaluate the order parameter from polarized transmitted light, we used the dichroic ratio as reported in Equation (1) when a transition moment is parallel to the principal axis of the molecules [24]:

(1) $$S=\frac{A_{∥}-A_{⊥}}{A_{∥}+2A_{⊥}}$$

where $A_{⊥}$ and $A_{∥}$ are the absorbances of the cell containing the molten monomers-mix, having respectively the director n (defining the average direction of the molecular long axes) perpendicular or parallel to the direction of the laser polarization. The absorbance was calculated as log_10_(1/T).

The birefringence of the molten mix was investigated by setting up a polarizer before the power detector and measuring the transmission when the rubbing direction of the cell was perpendicular, parallel or tilted by 45 degrees with respect to the direction of the laser polarization. (Supplementary Figure S1).

#### 2.2.4. Preparation of the Cell Reactor

For the photopolymer synthesis, cells were prepared, made of two glass slides and a plastic spacer. Each glass was dipped for 30 s in a solution of 1% elvamide in methanol (*w*/*w*) and dried at 160 °C for 1 h. Finally, the elvamide layers were rubbed automatically using an automatized home-made machine. The rubbed sides of the two glasses were faced towards each other in antiparallel mode (in respect to the rubbed direction) and spaced only by means of a 50 µm-thick kapton layer to get a cell reactor (Supplementary Figure S2).

#### 2.2.5. Synthesis of the Photo-Mobile Polymer Films

The PMP films were prepared using a mixture of LC monomers proposed by Lahikainen et al., 2018 [51]: 53 mol% of LC monomer 4-Methoxybenzoic acid 4-(6-acryloyloxyhexyloxy)phenyl es-ter (MAPE), 18 mol% of LC monomer 4[4[6-Acryloxyhex-1-yl)oxyphenyl]carboxybenzonitrile (AOCB), 22 mol% of di-acrylate crosslinker 1,4-Bis-[4-(6-acryloyloxyhexyloxy)benzoyloxy]-2-methylbenzene (AOBM), 6 mol% of azo crosslinker 4,4′-Bis[9 -(acryloyloxy)nonyloxy]azobenzene (A9ZA9) (Synthon Chemicals), and 1 mol % of photoinitiator Bis(2,4,6-trimethylbenzoyl)-phenylphosphineoxide. In brief, the reaction mixture was dissolved in dichloromethane and heated up at 70 °C until all the solvent was removed. Then, the reaction mixture was left to recrystallize in the fridge overnight until it was a compact powder.

The reaction cell was heated at 100 °C and the mixture infiltrated in the reactor cell by capillarity (Supplementary Figure S2). After the infiltration, the sample was moved onto a second hotplate set at the nematic temperature (50 °C) and photopolymerized for 1 h (30 min each side) using a UV LED lamp light λ = 400 nm (12 mW/cm^2^) and then left for 24 h at 50 °C.

This process was carried out both for bare PMPs and composites (ZnO: 6% *w*/*w*). For the preparation of ZnO-doped PMPs, the nanoparticles (100 nm) were added to the reaction mixture before the infiltration.

Undoped PMPs were labeled as Azo-LC-PMP, while ZnO-doped PMPs were labeled as Azo-LC-PMP(6%_Z).

To understand if our procedure was reproducible, the PMPs were prepared following two different protocols. In the first, the LC mixture and the nanoparticles were dry mixed firstly with a spatula and then with a vortex to facilitate the dispersion. The second approach involved the addition of DCM to the LC + ZnO mixture and then letting it dry as usual. Both approaches showed the same results.

#### 2.2.6. UV/vis Spectral Characterization (Absorbance, Transmission, Degradation)

Spectral characterization in UV/vis of the PMP films (Thickness ≈ 50 µm) was performed using the UV/VIS Spectrophotometer JASCO V-650 (accuracy 0.5 nm, range 190–850 nm, Oklahoma, OK, USA). Both total percentage transmittance T (%) and total percentage reflectance R (%) were measured using the integrating sphere JASCO ISN-722 (inside diameter 60 mm, range 200–870 nm).

The PMPs were further characterized to understand if the nanoparticles would reduce the decay time of the cis isomer of azobenzene [29]. The PMPs were irradiated for 5 and 60 min using a UVA lamp with peak wavelength at 400 nm with a power density of 12 mW/cm^2^. Then, the PMPs’ absorbance was measured after 1 and 24 h. Subsequently, a background film was prepared with all the components except A9AZ9 in order to exclude the contribution of the other LCs and the aliphatic chains.

The absorbance was calculated using the optical relation A (%) = 100 − T (%) − R (%).

#### 2.2.7. Thermographic Measurement

Thermographic measurements of the PMP film during and after laser irradiation were performed using the LWIR camera AVIO TVS 500 (spectral range 8–14 μm, FPA, 320 × 240, VOx microbolometer, Temperature resolution ~ 0.05 K, Yokoama, Japan) mounting a standard 22 mm lens. For temporal thermal trends the images were recorded with a frame rate of 20 Hz. Emissivity of the film was set to 0.93. All measurements were realized at a laboratory temperature of 23 °C and humidity of 50%.

#### 2.2.8. Atomic Force Microscopy (AFM)

The AZO-LC-PMP-Z 6% samples were morphologically characterized by atomic force microscopy. Analysis was carried out using the Veeco Dimension Digital Instruments Nanoscope IV (Plainview, NY 11803, USA) apparatus in the tapping mode configuration.

#### 2.2.9. Scanning Electron Microscopy and Energy Dispersive X-ray Analysis (SEM-EDX)

The microstructural analysis of the PMPs was performed by SEM. The SEM-EXD analyses of the gold evaporated PMP films were carried out by using a field emission scanning electron microscope (FEG-SEM, Leo 1530 Gemini by Zeiss, Oberkochen, Germany) with an operating voltage of 10–12 kV.

#### 2.2.10. Wide Angle X-ray Diffraction (WAXS)

Wide angle X-ray diffraction was used to analyze the crystal structure of samples (monomer powders and polymer films). The diffraction patterns were obtained with an automatic diffractometer (Philips X’PERT MPD) with Cu-Kα radiation (λ = 1.54056 Å) in different configurations. A θ–2θ configuration was used for the monomer while the analyses of polymer samples were carried out in the thin-film configuration by fixing θ at 2°. For both types of measurements, 2θ was changed from 5° to 60°, with a step of 0.05° and 5 s of acquisition for each point.

#### 2.2.11. Polarized Optical Microscopy

Olympus BX60 optical microscope (Tokyo, Japan) with crossed polarizers system and a magnification of ×10 was used to characterize both polymer films and monomers. The images of monomers in mesophase were taken at 50 °C by use of the temperature-controlled Linkam LTS120 stage.

#### 2.2.12. Dynamic Mechanical Analysis (DMA)

Dynamic mechanical analysis was performed using a Triton Technologies dynamic mechanical analyzer (now Mettler Toledo DMA1, Columbus, OH, USA) in tension mode. Mechanical properties of the polymer films (5 mm (length) × 5 mm (width) × 0.06 ± 0.005 mm (thickness)) were measured from −20 °C up to breaking, at a heating rate of 3 °C min^−1^ with an applied frequency of 1 Hz. Samples were preloaded with 0.5 N stress to ensure sample stiffness during analysis [52,53].

#### 2.2.13. Traction Ability

The traction ability of the PMPs was measured using the Instron universal testing instrument (model No. 5543A, Instron Engineering Corp., Norwood, MA, USA) equipped with a 1 kN cell. The samples were prepared as cantilevers with dimension of 30 × 20 mm or 20 × 15 mm. Once positioned between the clamps in traction mode, the sample was hit with a 445 nm laser with a spot of 5 × 1 mm. The power density in the area hit with the laser was 2,4 or 1 W/m^2^.

#### 2.2.14. Bending and Speed Characterization of PMPs

To study the dynamic response of the PMPs, both bare and doped Azo-LC-PMP were cut as cantilevers (5 mm × 1 mm) and irradiated at 457 nm with a 100:1 polarized laser. The laser wavelength was chosen according to the absorbance measurements carried out on the film and reported in Supplementary Figure S3 [27]. The set-up was composed of a neutral density filter, a retarder waveplate (λ/2), a focusing lens and a sample holder mounted on a 3D translator (Supplementary Figure S4).

The movements of the cantilevers were recorded at 60 fps. The videos have been unpacked using VLC (3.0.16) with the same frame velocity. The bending angle was measured considering the initial position of the cantilever with respect to its maximum bending. The angle was converted first to radian, then to arc length (mm), and the speed calculated in m/s. The seconds were derived by considering the number of frames in which the cantilever arrives to its maximum bending. Every speed point indicates the average bending speed during the path until the maximum bending.

## 3. Results and discussion

### 3.1. Thermal and Optical Properties of the Monomer Mixture

**Figure 1:**
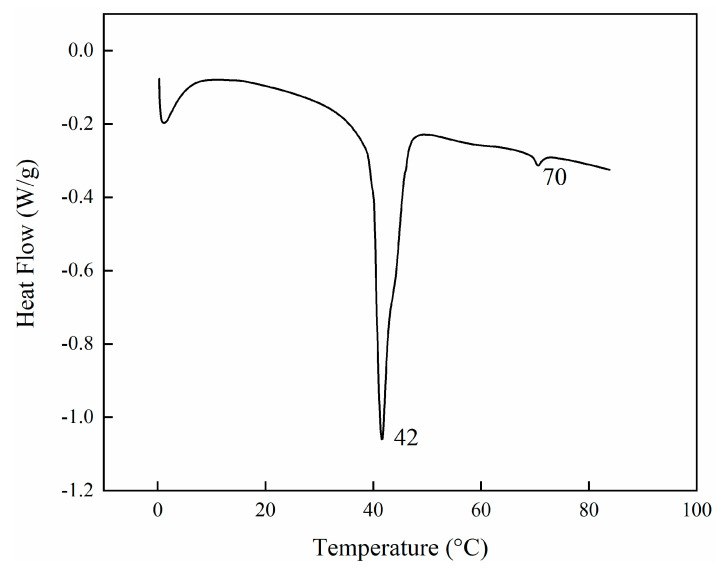
DSC profiles of LC monomer mixture melting when the initiator is absent. The thermogram corresponds to the first heating and shows two main endothermic transitions, the first at 42 °C and the second one at 70 °C.

Before any polymerisation attempts, the thermal behaviour of the LC mixture was studied by using a DSC. The thermogram showed the first endothermic peak at 42 °C (72 J/g) and the second at 70 °C (1.3 J/g) (Figure 1). The first and strongest peak corresponds to the overlapping of different phenomena because of the destruction of the crystalline phases of the monomers. In contrast, the second peak corresponds to the formation of an isotropic phase in the mixture. Since the LCs were a complex mixture, we could speculate that the nematic phase could be reached anywhere between the two endothermic transitions. Subsequently, to have a better understanding of the nematic order of the mixture, the order parameter (S) was calculated. This is a quantitative evaluation that would help us to understand the optimal conditions to have the mixture in a nematic state. If the transition moment related to the absorption is oriented parallel to the molecular long axis, the order parameter S can be calculated using the Equation (1).

**Figure 2:**
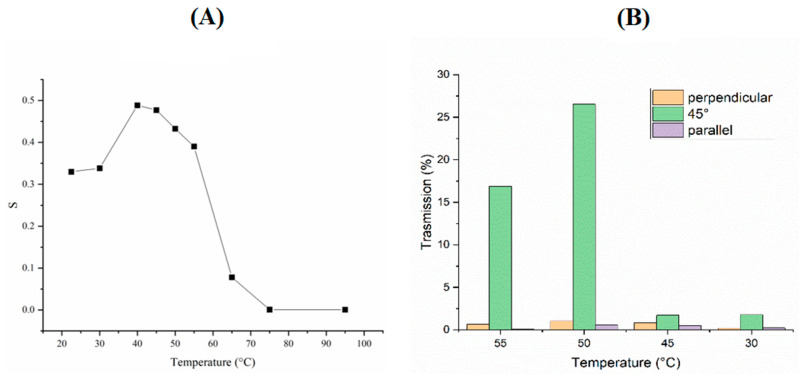
Order parameter experiment. (**A**) S calculated from transmission measurements vs. temperature with laser wavelength at 457 nm. (**B**) Transmitted light vs. temperature and for different orientations of light polarization with respect to the direction n (laser wavelength at 633 nm).

Two different polarized lasers were used with wavelengths of 457 nm and 633 nm. The laser at 457 nm was used to measure the orientational order of the mixture, showing a value of S = 0.5 was generally found (in literature the S is reported between 0.3 and 0.7) [54] (Figure 2A). Unfortunately, from these measurements it was only possible to define a range in which the mixture was in nematic state, between 40 and 60 °C (Figure 2A). In order to better define the range of the nematic phase, the transmittance at various temperatures was measured using the 633 nm laser. In particular, the measurements were taken in three different configurations when the director n  was: (i) parallel to the analyzer, (ii) parallel to the polarizer and (iii) tilted by 45 degrees (the scheme of the setup is shown in supplementary Figure S1). From Figure 2B, it is clear that at 50 °C the mixture is at its highest order. In fact, the transmitted light has a maximum due to the ability to rotate the polarized light.

The monomers mixed with the nanoparticles could not be analysed due to the phase separation. In fact, because of the length of the experiment, the nanoparticle would phase separate because of the effects of gravity.

### 3.2. Structural and Optical Characterizations of Photo-Mobile Polymers

After the monomer infiltration, the PMPs were synthesized in rubbed reactor-cells, peeled off and characterized. As a preliminary step, the crystalline structures of bare and ZnO doped PMP samples were studied using X-ray diffractometry. The diffractograms of the PMP films were collected and compared with those of the starting monomers. As shown in Supplementary Figure S5, all the monomers are crystalline, while their spectra are complex and characterized by many crystalline reflections. The main crystalline peaks are centered at 14.9° and 24.5° for A9zA9, and at 19.7° and 21.5° for the MAPE. The ACOB and AOBM present notably higher peaks than the others, respectively, at 25.3° and 20.4°.

**Figure 3:**
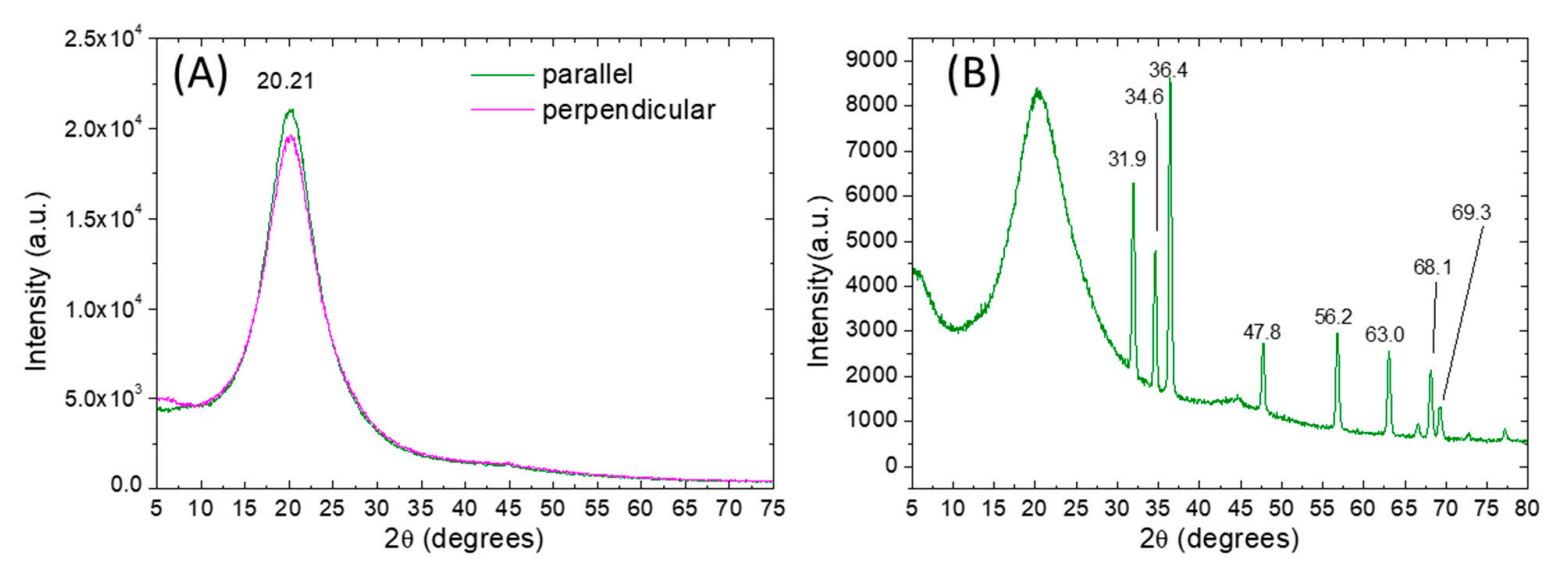
X-ray diffraction patterns (Cu Kɑ) of undoped AZO-LC-PMP (**A**) and doped AZO-LC-PMP-Z6% (**B**). For the undoped film the spectra are obtained by orienting the sample in two different ways (with the rubber direction parallel or perpendicular to the X-ray source).

Crystalline reflections of the starting monomers are not visible in the diffractograms of bare (Azo-LC-PMP) and doped PMP (Azo-LC-PMP(6%_Z)) films showing a complete polymerization for both formulations (Figure 3A,B). Indeed, independently from the orientation of the samples, all polymer spectra present a large peak at about 2θ of 22° associated with polymer chains in an amorphous state. Moreover, for Azo-LC-PMP(6%_Z) the amorphous spectra of the polymer were partially overlapped with the many, strong and narrow crystalline peaks of the ZnO nanoparticles. The peaks were centered at 31.9°, 34,6°, 36.4°, 47.8°, 56.2°, 63.0°, 66.6°, 68,1°, 69,3° corresponding to the lattice planes (100), (002), (101), (102), (110), (103), (112), (112) and (201) of the wurtzite structure of ZnO.

**Figure 4:**
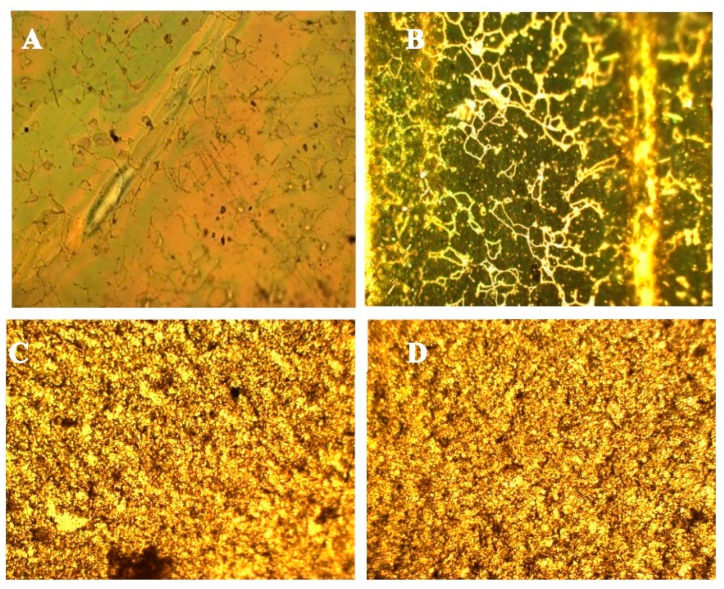
Optical images of Azo-LC-PMP (**A**,**B**) and doped PMP (**C**,**D**) obtained by polarized light microscope measured by putting the respective samples at 45 degrees (**A**,**C**) or parallel to one of the two polarizers (**B**,**D**).

Later, the PMPs were analysed with a polarized light microscope to find out if the nematic phase was stored after polymerization. The objective used was 10×, and the film was set both at 45 degrees and parallel to one of the two polarizers. The Azo-LC-PMP showed a well-defined property of rotation of polarized light (Figure 4A,B). This was confirmed by measuring the power of the light observed when the samples were rotated at different angles with respect to the two polarizers. A maximum transmission was measured when the cell was tilted of 45 degrees (Table 1). This result is an indication that our procedure allowed the synthesis of crosslinked films that have orientational ordering of the LC moieties.

For doped PMPs, no nematic alignment was detected. When the birefringence was tested, no significant difference was noted between the various configurations and formulations (Figure 4C,D, Table 1). This result shows that the ZnO-doped PMPs did not show orientational organization.

Together with polarized microscopy, in order to investigate the particles’ organization inside and outside the PMPs, SEM and AFM microscopy were performed.

**Figure 5:**
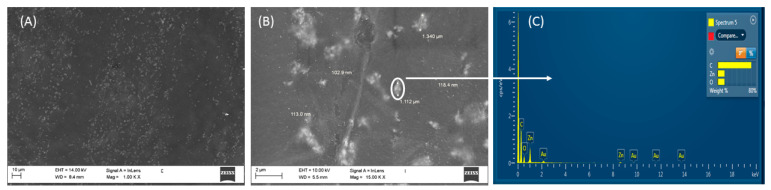
SEM images at different magnification of Azo-LC-PMP-Z6% (**A**,**B**) and an example of qualitative EDX analysis (**C**) carried out on the sample in correspondence of the aggregate indicated in B.

As highlighted by SEM (Figure 5) and standard optical microscopy (Supplementary Figure S6) aggregates (of size about 1.5–2 μm) are clearly visible on the surface of the doped PMP films at low magnification. The distribution of the aggregates seems not to be random. In fact, a higher concentration of them was observed along the rubbing direction (Figure 5A,B). The formation of particle trails of various sizes and lengths between the various clusters was detected both in the polymerised PMP and in the monomer melt (Supplementary Figure S7). The presence of these big aggregates suggests that a partial uncontrolled aggregation process has happened during the polymerization. It will be an object of further study in the future to minimize the occurrence of this process and improve the uniform dispersion of the ZnO in the samples.

SEM analysis at a higher magnification (Figure 5B) highlights the complex structures of the aggregates that consist of a collection of smaller nanoparticles as shown in Figure 5B. The qualitative analysis of the aggregates, carried out by EXD, confirms the presence of atoms of Zn and O, not detected in the adjacent areas.

From Figure 5B, it is also possible to observe the presence of a large number of smaller ZnO particles with diameters lower than 110 nm. The distribution of these particles was investigated more deeply by AFM.

**Figure 6:**
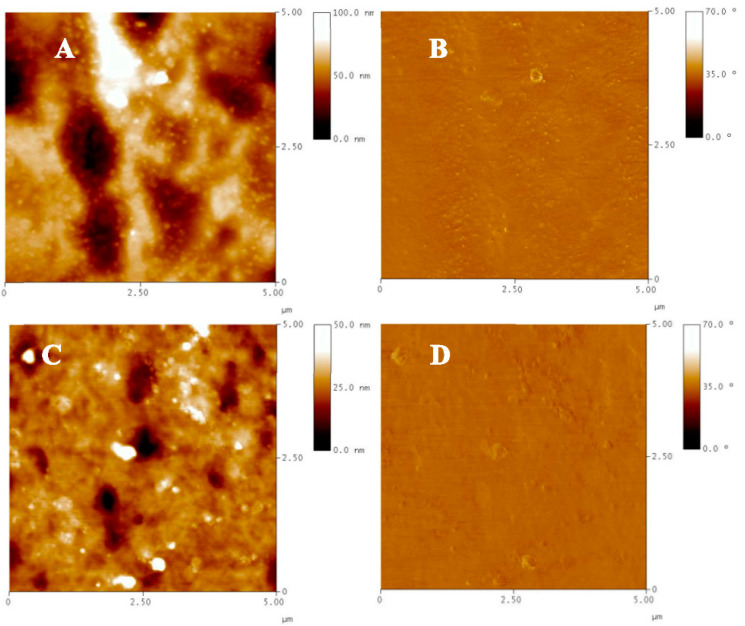
Topography and phase AFM images of ZnO-doped PMP (**A**,**B**) and bare PMP (**C**,**D**) films detected by scanning 5 × 5 µm^2^-sized areas.

Figure 6 shows topographic and phase contrast AFM images of the ZnO-doped PMP and bare PMP films. Topographic imaging of the composite-based sample clearly displays ZnO nanoparticles dispersed in the polymer matrix, almost uniformly distributed on the whole scanned area (Figure 6A). The root-mean-square roughness (Rq) valuated on the 5 × 5 μm^2^ surface gave a result equal to 14.02 nm. As expected, the AFM analysis of the undoped PMP, studied as a comparison, shows a flatter surface characterized by the Rq value estimated to be equal to 5.11 nm. In addition, phase images are also shown to uncover further details of the investigated morphologies: the phase, basically produced by the changes in adhesion between the tip and the sample surface, confirms nanostructures only with ZnO-doped PMP film (Figure 6B) and some of them are covered by (due to phase contrast generated by topographic variations) and others emerge from (due to phase generated by both topographic and compositional variations) the surrounding polymer. For the bare PMP sample, the phase image only reflects the surface topography and its inhomogeneities.

Three-dimensional imaging (Supplementary Figure S8) provides an informative view of the top surface of the doped PMP film studded with NPs.

The nanostructures observed on the surface of the composite-based film are organized as nanoaggregates with an estimated average size of about 100 nm, and mostly as single nanoparticles having an average diameter of 40–50 nm. The latter are more noticeably distinguishable by zooming the observation up to 2 × 2 μm^2^ areas (Supplementary Figure S9A). By imaging larger areas, the nanostructures’ distribution on the surface was highlighted. Independently of the poor surface homogeneity, it appears that the nanostructures are uniform along the rubbing direction (vertical direction of the 10 × 10 μm^2^-sized image) (Supplementary Figure S9B).

### 3.3. Thermomechanical Properties of PMPs

**Figure 7:**
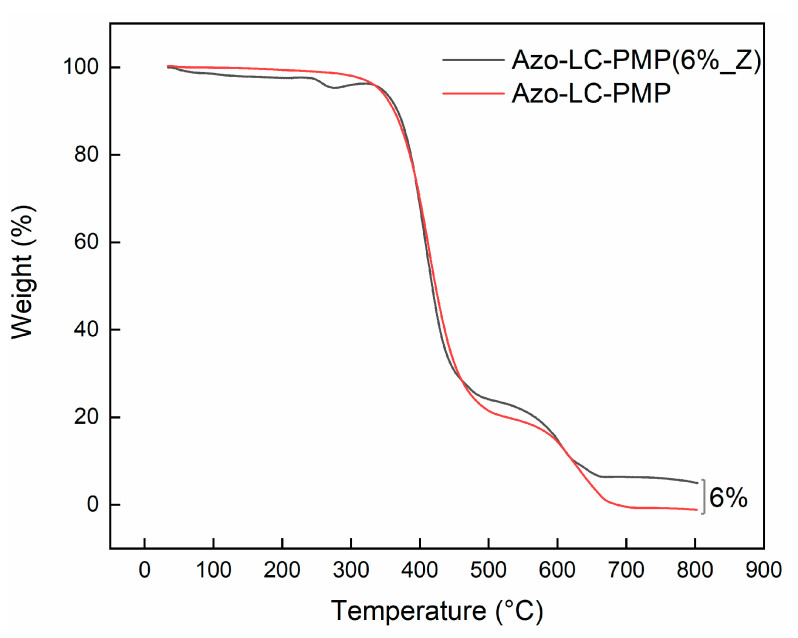
Thermogravimetric analysis of bare and doped PMP film, showing the degradation as a function of the temperature.

After the morphological analysis, the PMPs were characterized for their thermomechanical properties. At first, a scan TGA experiment was performed to study the thermal stability of the samples (Figure 7).

Concerning the thermal stability of the samples, degradation of the bare PMP starts at about 367 °C, about 20% of weight loss occurred up to 404 °C, then subsequent heating leads to complete degradation of the material in the range of 404–700 °C. The presence of ZnO nanoparticles did not alter the thermal stability of the polymer in terms of degradability, since the onset degradation temperature of the nanocomposite is 369 °C and the temperature corresponding to the maximum weight loss of the first degradation step, $T_{d1}$, is 409 °C. The second degradation step was shifted towards a lower temperature in the doped sample compared to the plain polymer. As expected, there is no difference between the two samples except for the final weight at the end of the process. In fact, for the Azo-LC-PMP(6%_Z) there is 6% more residue because of the ZnO nanoparticles (Figure 7).

The Azo-LC-PMP DSC-thermogram displayed a glass transition ($T_{g}$) at 36.4 °C. Glass transition of the ZnO nanocomposite, centred at 40.7 °C, is slightly shifted towards higher temperatures (Supplementary Figure S10). The presence of well-dispersed ZnO nanoparticles could reduce the segmental mobility of the polymeric chains at the interface, thus shifting the $T_{g}$ of the nanocomposite.

**Figure 8:**
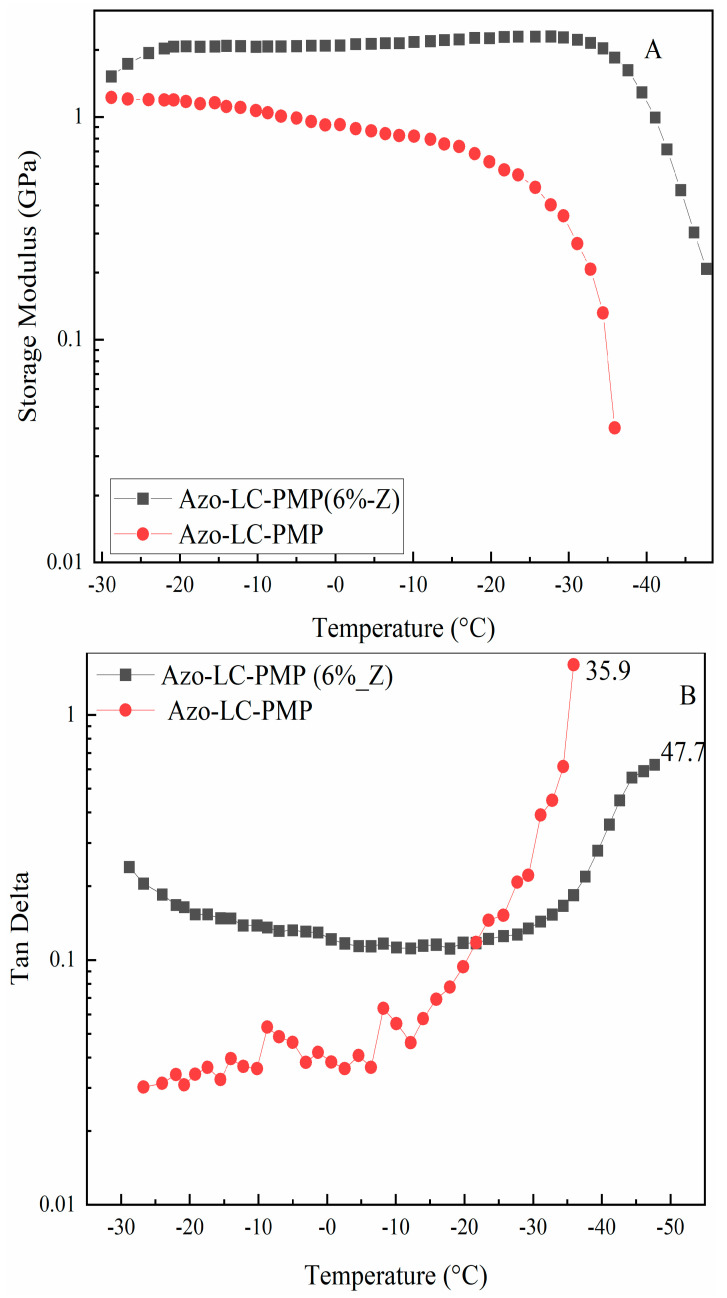
Dynamic mechanical analysis of Azo-LC-PMP amd Azo-LC-PMP(6%_Z). (**A**) Comparison of storage modulus of the two formulations (**B**) TanDelta comparison of the Azo-LC-PMP(6%_Z) and Azo-LC-PMP.

Besides this, in order to understand if ZnO nanoparticles would affect the thermomechanical response of the materials, the PMPs were tested using a DMA in a gradient of temperature. When the storage modulus (G’) of the two materials was compared, a higher G’ was recorded for Azo-LC-PMP(6%_Z). In fact, it was more stable temperature-wise and twofold higher for Azo-LC-PMP (6%_Z) compared to Azo-LC-PMP (Figure 8A). Increasing the temperature from 0 °C, the bare PMP storage modulus began to decrease as the temperature approached 35 °C. For the Azo-LC-PMP(6%_Z) the storage modulus remained stable around 2 GPa up to the rubbery zone in the same temperature range. The glass transition temperature increases for the composite material, as shown by the TanDelta (calculated as the ratio between storage and loss moduli) (Figure 8B) and confirmed in the DSC (Supplementary Figure S10). The $T_{g}$ increase is most likely partially because of nanoparticles in high concentration [55]. In fact, as mentioned above, the chain end of the polymer would be hindered by the particles and would have difficulties vibrating during the DMA experiment.

**Figure 9:**
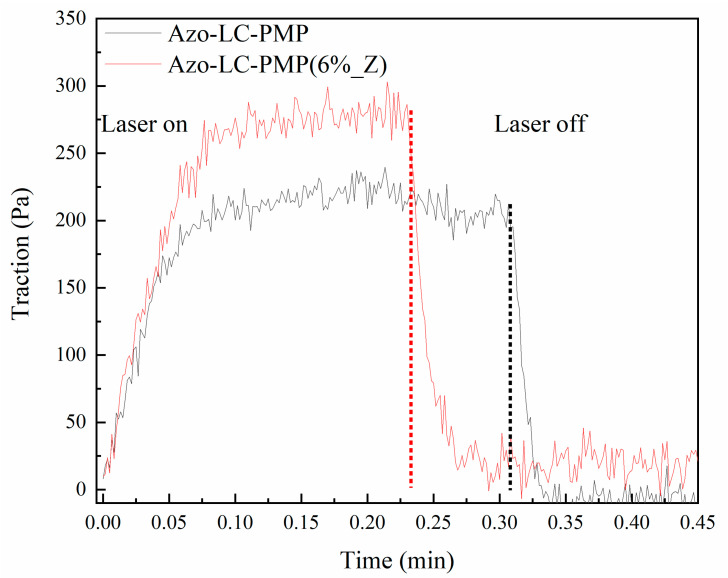
Traction ability of the PMPs when irradiated by the laser. The power density was 2.4 W/cm^2^. The laser starts to irradiate the sample at 0.0 min and stops on the dotted line.

Another result that showed the superior properties of the composite PMP compared to the bare version was the measuring of the traction ability of the films reported in Figure 9. The PMPs were irradiated while clamped to a dynamometer. The Azo-LC-PMP(6%_Z), when irradiated with 2.5 W/cm^2^, was able to express a higher force when at plateau (275 Pa) compared to the bare film (200 Pa). Furthermore, when the PMPs were irradiated with a laser power as low as 1 W/cm^2^, only Azo-LC-PMP(6%_Z) was able to produce some force. It is important to note that such an experiment was performed irradiating PMPs on a small area (0.05 cm^2^) compared to the total area of the PMPs (x cm^2^).

### 3.4. ZnO Nanoparticles Effect on Azo-LC-PMP/Light Interaction

**Figure 10:**
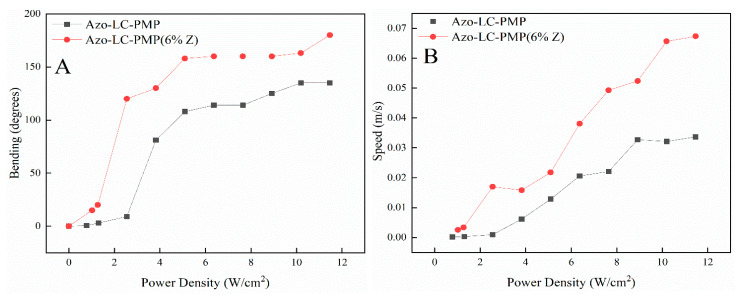
Comparison between the maximum bending (**A**) and speed (**B**) of Azo-LC-PMP and ZnO doped AZO-LC-PMP films. The graphs show the increased efficiency of the doped PMPs in terms of bending and speed.

The bare PMPs were optimized as previously described and then optically characterized to investigate their performance by a test of self-vibration as described in the literature [5,51]. The cantilever-shaped PMPs were irradiated with a 457 nm laser with a laser-spot with a radius of 0.5 mm. The power density (area = 0.008 cm^2^) was modulated in a range between 1 and 10 W/cm^2^. The power threshold to bend the film synthesized at 50 °C (Figure 10A) was 1 W/cm^2^ and the self-oscillating threshold was 7.5 W/cm^2^ (Supplementary Figure S11A). To show that the optimized Azo-LC-PMP, cured at a nematic temperature (50 °C), performed better than formulations prepared at higher temperatures, it was compared with one prepared at 60 °C (Supplementary Figure S11B). Indeed, already at 4 W/cm^2^ the speed of Azo-LC-PMP was 7.8-fold higher than the one polymerized at higher temperature (Supplementary Figure S11B). These results confirm the importance of studying the thermal and optical properties of a new monomer mixture in order to detect its nematic temperature optimizing the long-range order in bare PMP films.

Afterward, the bending properties of Azo-LC-PMPs (manufactured at 50 °C) were studied to understand if the nanoparticles would affect their light response. The polymer films were tested for their bending speed, bending capability and response threshold to light irradiation. The Azo-LC-PMP(6%_Z) performed better than Azo-LC-PMP both in terms of maximum bending angle and bending speed (Figure 10). The power threshold to move the doped sample was again 1 W/cm^2^. Interestingly, at 1 W/cm^2^, bending of the composite film was eight times higher than the Azo-LC-PMP film (Figure 10A). At this power density, the speed was nine times higher. The speed difference decreases with an increase in the power density. At the maximum measured power density (12 W/cm^2^) the doped PMPs had a speed about two times higher (Figure 10B).

Thermal and spectral characterization were performed to understand the nature of the observed effect.

**Figure 11:**
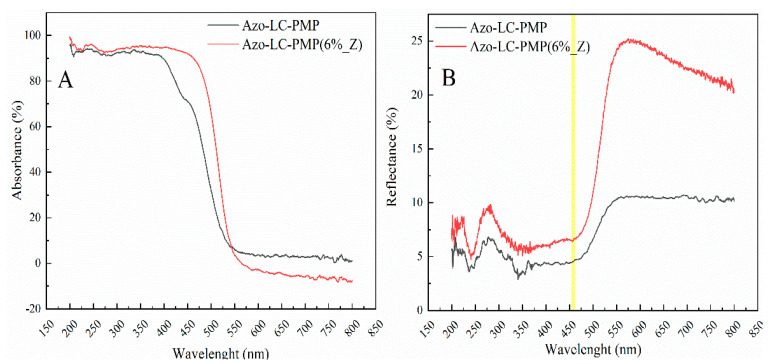
Comparison between the spectra of (**A**) absorbance, and (**B**) reflectance of both Azo-LC-PMP and doped Azo-LC-PMP Z.

In Figure 11A,B the UV/vis characterizations of the bare and doped PMPs are reported. Both Azo-LC-PMP and Azo-LC-PMP(6%_Z) present an absorbance that goes approximately up to 95% in the wavelength range of 300–400 nm. In contrast, for Azo-LC-PMP(6%_Z), an increase of absorption was recorded with a range between 400 nm and 500 nm (Figure 11A). For example, the absorbance at 457 nm shows an increase of 40% compared to Azo-LC-PMP. This probably allows the PMP to absorb higher amounts of radiation in a region other than UV (Figure 11A). The increase in absorbance is probably due to the high reflectivity of the ZnO nanopowder [56] embedded inside the PMP. In fact, these kinds of nanoparticles are also used in scattering layers for different applications [56].

The total reflectance at 457 nm shows an increase of 2.5% for Azo-LC-PMP(6%_Z). When higher wavelengths are considered (550–800), the reflectance shows a noticeable increase, confirming that particles also appear on the PMP surface as shown with the SEM and AFM (Figure 5 and Figure 6).

Additionally, the spectral analysis showed another interesting behavior of Azo-LC-PMP(6%_Z) related to the extinction of the cis isomer of the azobenzene. In fact, when the ZnO is embedded into the PMP matrix, the *cis* isomer isomerizes back into *trans* in a shorter time. After an irradiation of 15 min with the UV lamp and a dark period of 1 h, the concentration of cis isomers in the Azo-LC-PMP is 7% higher than in Azo-LC-PMP(6%_Z) (Supplementary Figure S3). This effect was previously attributed to an exchange of electrons between metal and azobenzene, when gold nanoparticles were embedded in the PMP [29,35].

**Figure 12:**
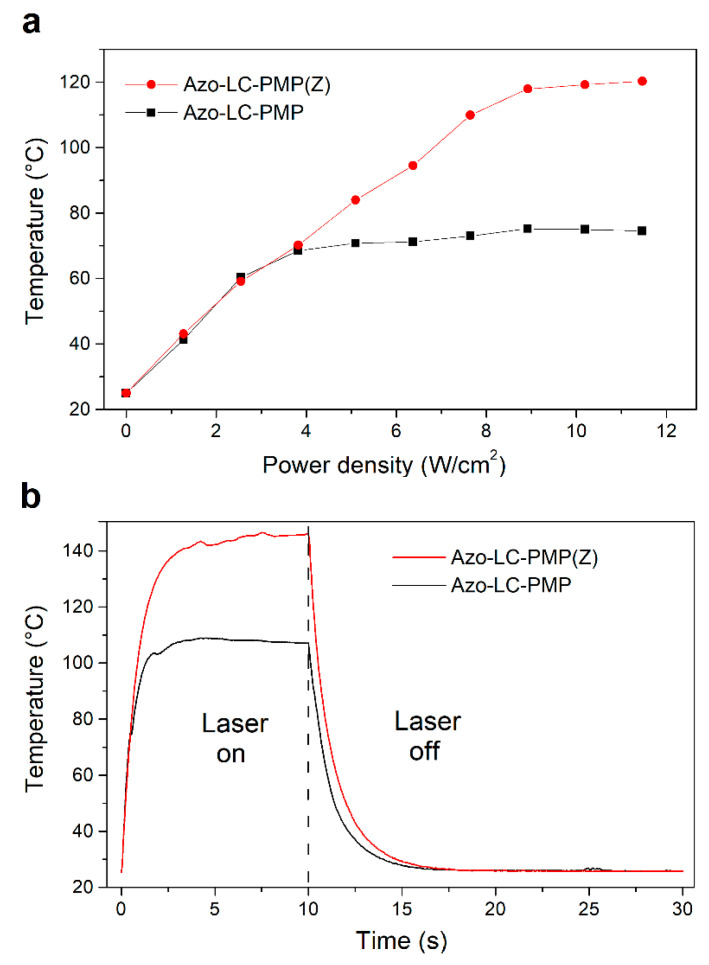
Thermographic measurements of the undoped (black color) and doped (red color) PMP film: (**a**) temperature at the equilibrium condition versus the power density of the laser irradiation, (**b**) temporal trend of the temperature during and after laser irradiation with a power density of 11.5 W/cm^2^.

The thermal behavior of PMP films both with and without ZnO particles under laser irradiation (λ = 457 nm) was investigated and compared by means of thermographic analysis. In Figure 12A, the temperatures measured for the two films at a density power irradiation ranging between 0–11.5 W/cm^2^ are reported. The temperatures refer to values achieved after 10 s of irradiation in the condition of thermal equilibrium. Each value represents an average estimated on a film area of 2 mm^2^.

The trend of the two films is similar up to the power density of about 3 W/cm^2^ (corresponding to a film temperature of about 65 °C). The effect of the particles up to these values appears negligible. At a higher power density, the effect of the ZnO particles appears clear. While the undoped PMP film shows a plateau around 75 °C, the temperature of the doped PMPs continues to increase linearly up to a maximum value of about 120 °C (11.5 W/cm^2^). This last result shows how the presence of ZnO particles favors the absorption of the radiation, thus increasing the heat absorbed by the PMP film, above a certain threshold (3 W/cm^2^).

Interestingly, comparing the temperature trend (Figure 12) of Azo-LC-PMP(Z) with its speed (Figure 10B), the two quantities seem to be correlated. This shows that heat plays an important role in improving the performance of the doped PMP.

In Figure 12B, the temporal response of the temperature of both doped and undoped film is shown when they are irradiated with the same laser source for 10 s and with a power density of 11.5 W/cm^2^. As expected, because of the metallic inclusions, the temperature rate of the doped PMPs is higher with respect to bare PMPs in both heating and cooling phases. During the heating phase, the rate is about 93 °C/s for the bare PMPs and about 107 °C/s for the doped one. In contrast, during the cooling phase, the rates are about 11 °C/s for undoped film and 16 °C/s for the PMPs with the ZnO. Moreover, the trend of the bare PMPs reaches a plateau at a temperature of 107 °C while the doped film reaches a maximum temperature of 146 °C. At those temperatures, the undoped film starts to burn while the doped film does not, indicating a possible dissipation effect due to the metal oxide.

As briefly mentioned, there is a correlation between the bending of the PMPs, their response to light stimuli, and induced thermal effects. In fact, when the temperature reaches the plateau for the Azo-LC-PMP (Figure 12A, 4 W/cm^2^), the bending has a constant value (Figure 10A, ~100 degrees) until a slight flex in the temperature is recorded (Figure 12A, 8 W/cm^2^) and the bending starts to increase again. When Azo-LC-PMP (6%-Z) is analyzed, the bending becomes constant (~150 degrees) at a higher temperature (90 °C) indicating that the presence of metallic nanoparticles would cause a thermotropic effect. This correlation is observed for the speed of the PMPs too. Our hypothesis is that an azobenzene photoisomerization equilibrium (*trans-cis-trans*) is reached. In fact, the laser induces the *trans* to *cis* isomerization and instead, heat induces the inverse process.

Further behavior worth noting is related to the linear increase of the PMP speed for the Azo-LC-PMP(6%_Z) (Figure 10A). In fact, the speed of the doped PMP is always higher, and it increases linearly. This is possibly correlated with an increase in temperature in the irradiated area, causing a softening of the polymer in that spot. This effect would help the bending and velocity without affecting much the mechanical properties (Figure 8A) that were improved because of the presence of ZnO.

## 4. Conclusions

A liquid crystal mixture doped with ZnO nanoparticles was characterized to evaluate its use in the synthesis of novel efficient PMPs. We demonstrated that our facile method for the fabrication of the PMPs was highly reproducible and allowed the formation of a distributed nanoparticles network in the film. The nanoparticles were present both as a fine dispersion and clusters and, interestingly, were distributed throughout the whole film and not only in the surface.

The addition of nanoparticles to the PMP improved the mechanical properties of the material. In fact, the G’ measured using a DMA was two times higher and more stable between −10 and 40 °C. Furthermore, the $T_{g}$ increased considerably.

Spectroscopic and thermal behavior of doped photomobile films was deeply investigated by means of UV-VIS spectroscopy and thermographic experiments. Focusing on a wavelength region (around 457 nm) where the absorption band of the undoped mixture start to decrease (≥70%), we demonstrated the possibility to increase light absorption, expanding the bandwidth in which the PMPs could properly work. Comparing doped and bare PMPs, it is clear that the improvement in efficiency in terms of light conversion into mechanical movement is due to the ZnO nanoparticles that strongly modify both the spectral and thermal properties of the fabricated films.

## Figures and Tables

**Table 1:** Transmissivity of bare and doped PMP when the director n was parallel to the polarizers or tilted by 45 degrees, with incident light power $P_{in}=2.5\;\mathrm{mW}.$

Sample Name	Parallel to Polarizer (μW)	Tilted by 45 Degrees (μW)	Parallel to Analyzer (μW)
Azo-LC-PMP	15.5	403	24.2
Azo-LC-PMP doped	0.92	0.62	0.45

## Data Availability

Not applicable.

## Supplementary Materials

The following are available online at https://www.mdpi.com/article/10.3390/nano11123320/s1, Figure S1: Setup for the measurement of transmitted light and birefringence. Figure S2: (Up) Preparation of glass reactor cells and polymerization. (down) Monomer and Polymer structure. Figure S3: (A) Absorbance of the azobenzene moieties embedded in the PMP, obtained by subtracting the contribution of the other elements in the PMP (B) Absorbance of azobenzene moieties in *trans* post irradiation, obtained subtracting the absorbance of the PMP pre-UV. Figure S4: Setup for characterization of PMPs light response. Figure S5: X-ray diffraction patterns (Cu Kɑ) of the starting monomer powders. Figure S6: Picture of melted monomer penetrated in the cell (left) and outside the cell(right). In both the pictures it is possible to see trails formed by the interaction of nanoparticles and LCs. Objective 10×. Figure S7. Aggregates and trails formed by nanoparticles. On the left a closeup picture (100×) of a cell-like structure in a Azo-LC-PMP(6%-Z) and on the right a Azo-LC-PMP (1%-ZnO) mixture showing the aggregates and trails (10×). Figure S8: 3D-topography AFM image of ZnO-doped PMP film detected by scanning 5 × 5 µm^2^-sized area. Figure S9: Topography AFM images of ZnO-doped PMP film detected by scanning 2 × 2 µm^2^ (A) and 10 × 10 μm^2^ (B) -sized areas. Figure S10: DSC Thermograms of Azo-LC-PMP and ZnO composite scans. Figure S11: (left) Self-Oscillating Azo-LC-PMP without ZnO nanoparticles (right) Bending speed (m/s) of Azo-LC- PMP Z polymerized at 50 °C and 60 °C. Showing the improved performance of the film when polymerized at the nematic temperature.
