# Supplementary material for: Photo-Responsivity Improvement of Photo-Mobile Polymers Actuators Based on a Novel LCs/Azobenzene Copolymer and ZnO Nanoparticles Network
Source: Nanomaterials (Basel). 2021 Dec 7;11(12):3320. doi: 10.3390/nano11123320 (PMC8705796; doi:10.3390/nano11123320)
Supplement: Supplementary file 1 [file nanomaterials-11-03320-s001.zip › nanomaterials-1476933-supplementary.pdf]

Supplementary Materials

Photo-responsivity Improvement of Photo-Mobile Polymers Actuators Based on a Novel LCs/Azobenzene Copolymer and ZnO Nanoparticles Network

Domenico Sagnelli [1,*], Marcella Calabrese [1,7], Olga Kaczmarczyk [1,2], Massimo Rippa [1], Ambra Vestri [1], Valentina Marchesano [1], Kristoffer Kortsen [3], Valentina Cuzzucoli Crucitti [4], Fulvia Villani [5], Fausta Loffredo [5], Carmela Borriello [5], Giuseppe Nenna [5,*], Mariacristina Cocca [6], Veronica Ambrogi [7], Katarzyna Matczyszyn [2], Francesco Simoni [1] and Lucia Petti [1,*]

1 Institute of Applied Sciences and Intelligent Systems of CNR, 80072 Pozzuoli, Italy; marcellacalabrese.97@libero.it (M.C.); olga.kaczmarczyk@pwr.edu.pl (O.K.); massimo.rippa@isasi.cnr.it (M.R.); ambra.vestri@isasi.cnr.it (A.V.); valentina.marchesano@isasi.cnr.it (V.M.); f.simoni@photomat.it (F.S.)
2 Advanced Materials Engineering and Modelling Group, Faculty of Chemistry, Wroclaw University of Science and Technology, Wybrzeze Wyspianskiego 27, 50-370 Wroclaw, Poland; katarzyna.matczyszyn@pwr.edu.pl
3 School of Chemistry, University of Nottingham, University Park, Nottingham NG7 2RD, UK; Kristoffer.kortsen@nottingham.ac.uk (K.K.);
4 Department of Chemical and Environmental Engineering, University of Nottingham, University Park, Nottingham NG7 2RD, UK; valentina.cuzzucolicrucitti1@nottingham.ac.uk (V.C.C.)
5 ENEA, Italian National Agency for New Technologies, Energy and Sustainable Economic Development, Portici Research Centre, Portici, I-80055 Naples, Italy; fulvia.villani@enea.it (F.V.); fausta.loffredo@enea.it (F.L.); carmela.borriello@enea.it (C.B.)
6 Institute for Polymers, Composites and Biomaterials of CNR, 80072 Pozzuoli, Italy; mariacristina.cocca@ipcb.cnr.it
7 Department of Chemical, Materials and Production Engineering, University of Naples Federico II,
80125 Naples, Italy; veronica.ambrogi@unina.it
* Correspondence: domenico.sagnelli@isasi.cnr.it (D.S.); Giuseppe.nenna@enea.it (G.N.); lucia.petti@isasi.cnr.it (L.P.)

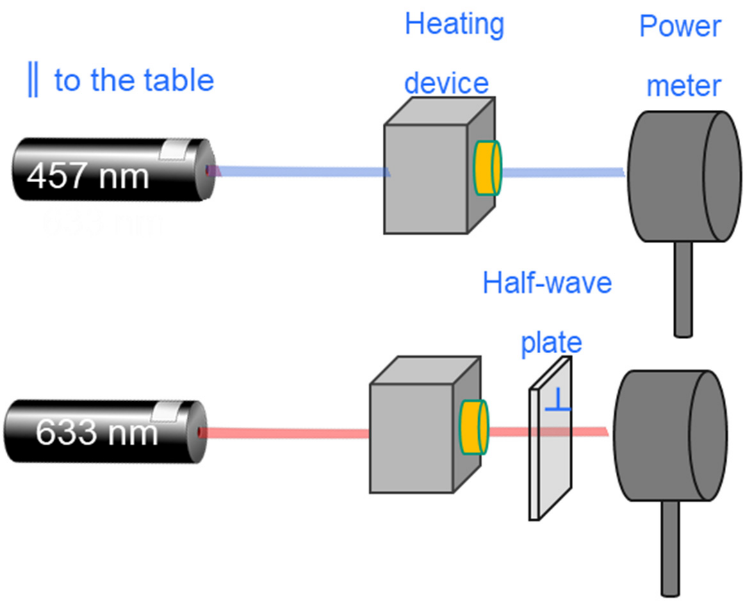

Figure S1. Setup for the measurement of transmitted light and birefringence.

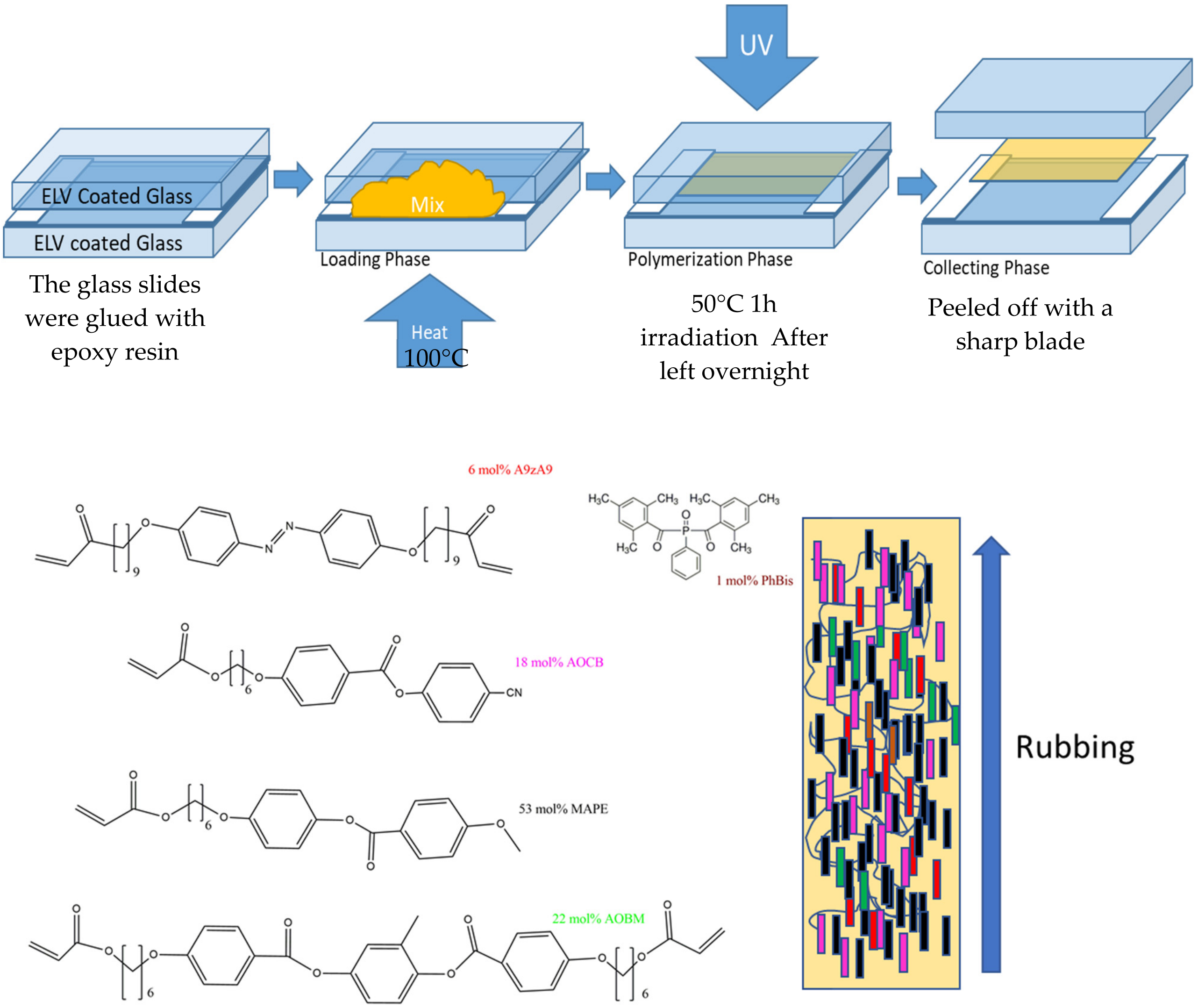

Figure S2. (**Up**) Preparation of glass reactor cells and polymerization. (**down**) Monomer and Polymer structure.

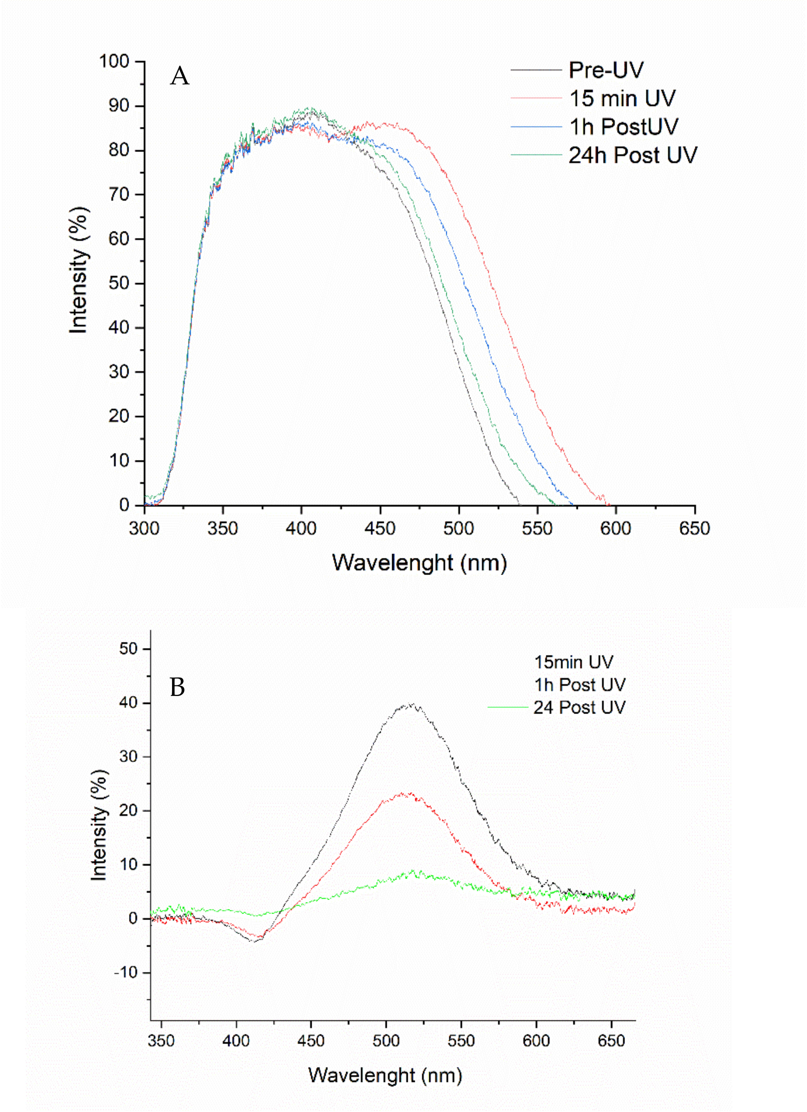

Figure S3. (**A**) Absorbance of the azobenzene moieties embedded in the PMP, obtained by subtracting the contribution of the other elements in the PMP (**B**) Absorbance of azobenzene moieties in *trans* post irradiation, obtained subtracting the absorbance of the PMP pre-UV.

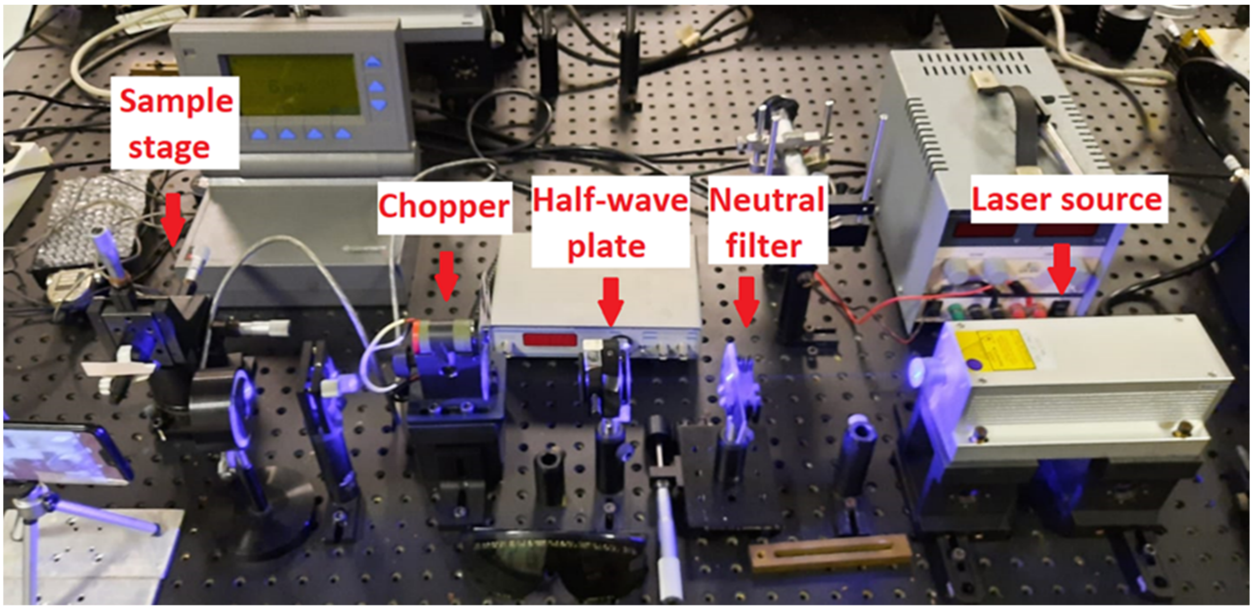

Figure S4. Setup for characterization of PMPs light response.

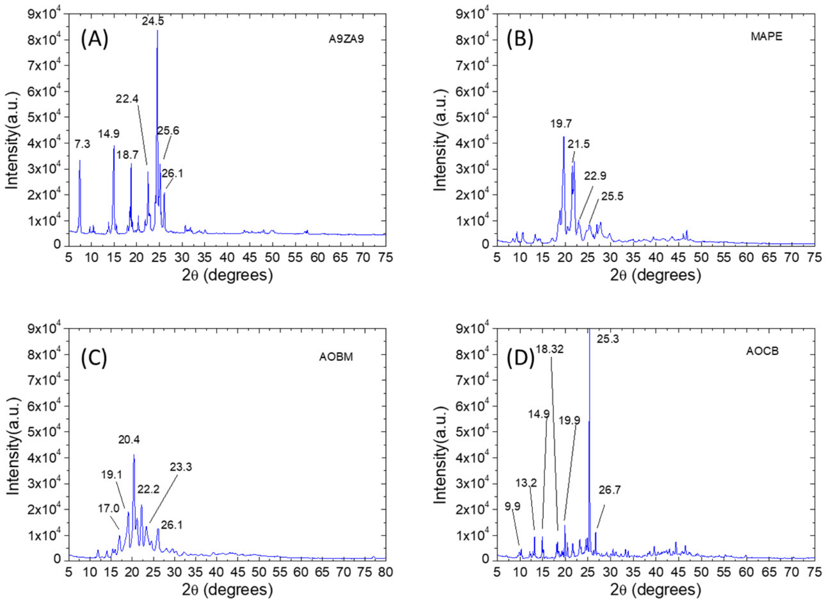

Figure S5. X-ray diffraction patterns (Cu Kα) of the starting monomer powders. (**A**) A9ZA9, (**B**) MAPE, (**C**) AOBM, (**D**) AOCB.

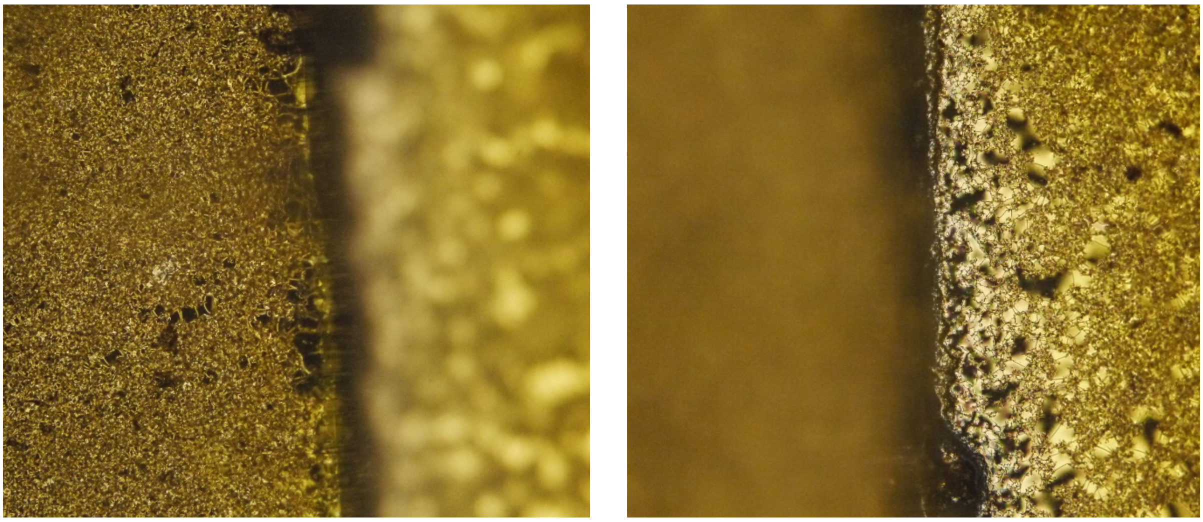

Figure S6. Picture of melted monomer penetrated in the cell (**left**) and outside the cell (**right**). In both the pictures it is possible to see trails formed by the interaction of nanoparticles and LCs. Objective 10x.

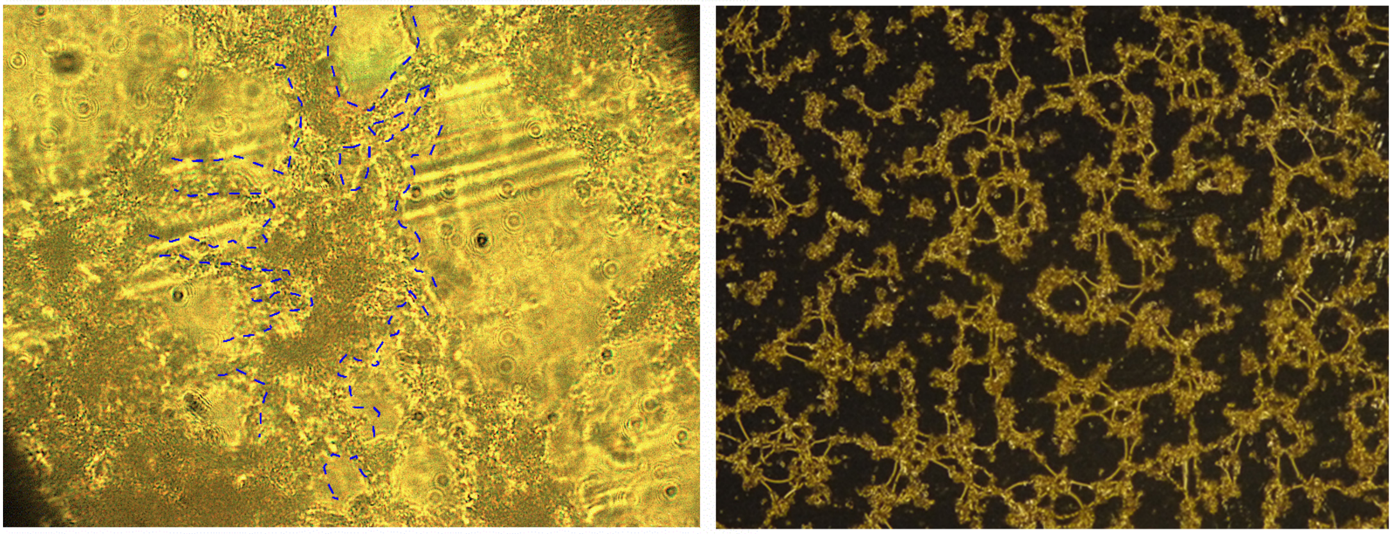

Figure S7. Aggregates and trails formed by nanoparticles. On the **left** a closeup picture (100x) of a cell-like structure in a Azo-LC-PMP(6%-Z) and on the **right** a Azo-LC-PMP (1%-ZnO) mixture showing the aggregates and trails (10x).

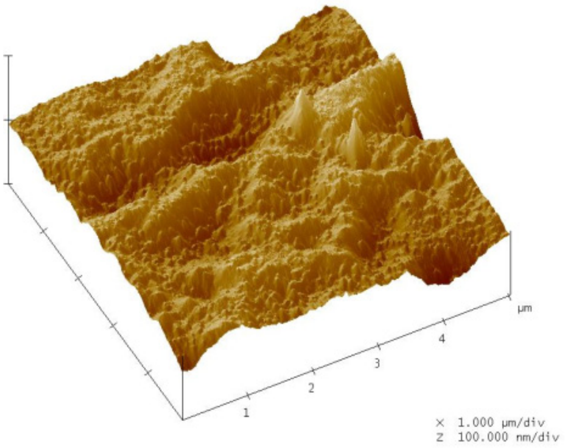

Figure S8. 3D-topography AFM image of ZnO-doped PMP film detected by scanning 5 × 5 μm^2-sized area.

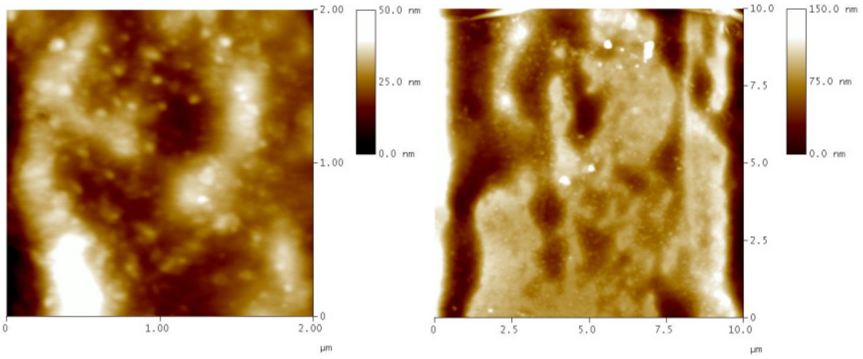

(**A**) (**B**)

Figure S9. Topography AFM images of ZnO-doped PMP film detected by scanning 2 × 2 μm^2 (**A**) and 10 x 10 m^2 (**B**) - sized areas.

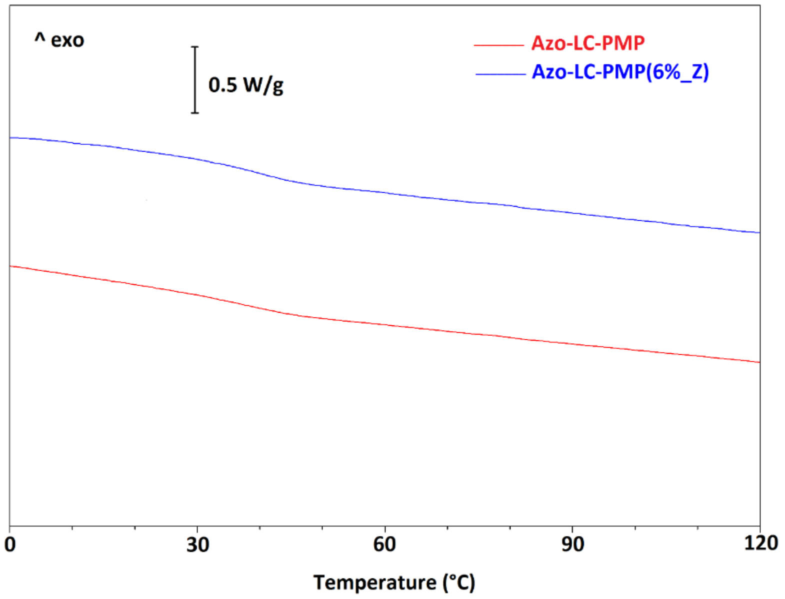

Figure S10. DSC Thermograms of Azo-LC-PMP and ZnO composite scans.

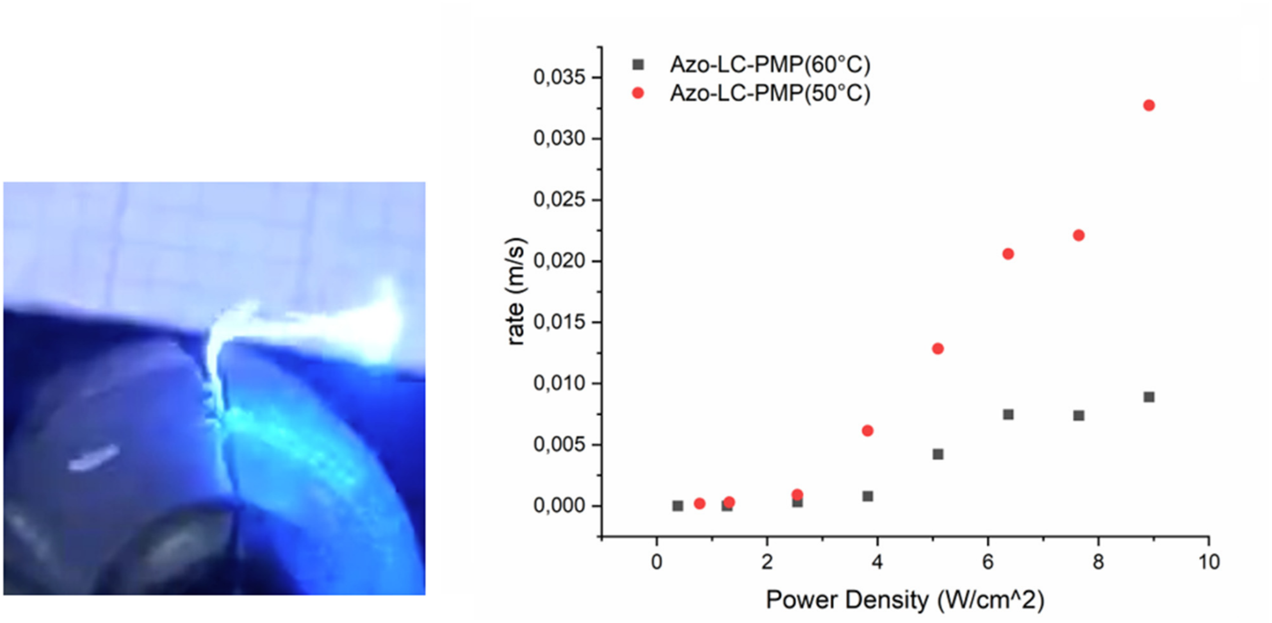

Figure S11. (**left**) Self-Oscillating Azo-LC-PMP without ZnO nanoparticles (**right**) Bending speed (m/s) of Azo-LC- PMP Z polymerized at 50 °C and 60 °C. Showing the improved performance of the film when polymerized at the nematic temperature.
